# Beyond Trikafta: new models to assess tissue dependent rescue of N1303K-CFTR

**DOI:** 10.3389/fphar.2025.1661417

**Published:** 2025-10-29

**Authors:** Iwona Pranke, Valeria Capurro, Benoit Chevalier, Emanuela Pesce, Valeria Tomati, Cristina Pastorino, Mairead Kelly-Aubert, Aurelie Hatton, Elise Dreano, Mariateresa Lena, Renata Bocciardi, Federico Zara, Stefano Pantano, Vito Terlizzi, Cristina Lucanto, Stefano Costa, Laura Claut, Valeria Daccò, Piercarlo Poli, Massimo Maschio, Benedetta Fabrizzi, Nicole Caporelli, Marco Cipolli, Sonia Volpi, Frederique Chedevergne, Laure Cosson, Julie Macey, Sophie Ramel, Laurence Weiss, Dominique Grenet, Laurence Le Clainche-Viala, Benoit Douvry, Bruno Ravoninjatovo, Camille Audousset, Aurélie Tatopoulos, Bénédicte Richaud-Thiriez, Melissa Baravalle, Guillaume Thouvenin, Guillaume Labbé, Marie Mittaine, Philippe Reix, Isabelle Durieu, Julie Mankikian, Stéphanie Bui, Thao Nguyen-Khoa, Karim Khoukh, Clémence Martin, Jennifer Da Silva, Paola De Carli, Carlo Castellani, Federico Cresta, Luis Galietta, Anne Guillemaut, Emmanuelle Girodon, Natacha Remus, Mattijs Bulcaen, Marjolein Ensinck, Miroslaw Zajac, Marianne Carlon, Jean LeBihan, Pierre-Régis Burgel, Isabelle Sermet-Gaudelus, Alexandre Hinzpeter, Nicoletta Pedemonte

**Affiliations:** 1 INSERM, CNRS, Institut Necker Enfants Malades, Paris, France; 2 Université Paris-Cité, Paris, France; 3 UOC Genetica Medica, IRCCS Istituto Giannina Gaslini, Genova, Italy; 4 Department of Neurosciences, Rehabilitation, Ophthalmology, Genetics, Maternal and Child Health (DINOGMI), University of Genoa, Genova, Italy; 5 UOSD CRR Fibrosi Cistica, Atri, Italy; 6 Department of Pediatric Medicine, Meyer Children’s Hospital IRCCS, Cystic Fibrosis Regional Reference Center, Florence, Italy; 7 Centro Hub Fibrosi Cistica, Azienda Ospedaliera Universitaria Policlinico G. Martino, Messina, Italy; 8 Department of Pediatrics, University Hospital “G Martino”, Messina, Italy; 9 Department of Pediatrics, Cystic Fibrosis Center, Fondazione IRCCS Ca’ Granda, Ospedale Maggiore Policlinico, Milan, Italy; 10 Department of Pediatrics, Cystic Fibrosis Regional Support Center, University of Brescia, ASST Spedali Civili Brescia, Brescia, Italy; 11 Institute for Maternal and Child Health-IRCCS “Burlo Garofolo”, Trieste, Italy; 12 Cystic Fibrosis Regional Centre, Unit of Emerging and Immunosuppressed Infectious Diseases, Department of Gastroenterology and Transplantation, Azienda, Italy; 13 Ospedaliero-Universitaria ‘Ospedali Riuniti’, Ancona, Italy; 14 Cystic Fibrosis Center of Verona, Azienda Ospedaliera Universitaria Integrata, Verona, Italy; 15 Cystic Fibrosis National Pediatric Reference Center, Pneumo-Allergologie Pédiatrique, Hôpital Necker Enfants Malades, AP-HP, Paris, France; 16 Centre de Ressources et de Compétence de la Mucoviscidose Enfants, Hôpital de Clocheville, Tours, France; 17 Centre de Ressources et de Compétence de la Mucoviscidose, CHU Pellegrin, Bordeaux, France; 18 Centre de Ressources et de Compétence de la Mucoviscidose Adulte, Centre de Perharidy, Roscoff, France; 19 Centre de Ressources et de Compétence de la Mucoviscidose Pédiatrique, CHU, Strasbourg, France; 20 Centre de Ressources et de Compétence de la Mucoviscidose, Hôpital Foch, Suresnes, France; 21 Centre de Ressources et de Compétence de la Mucoviscidose Pédiatrique, Hôpital Robert Debré, Paris, France; 22 Centre de Ressources et de Compétence de la Mucoviscidose Mixte, CHIC, Créteil, France; 23 Centre de Ressources et de Compétence de la Mucoviscidose, American Memoral Hospital, Reims, France; 24 Centre de Ressources et de Compétence de la Mucoviscidose, Institut Cœur Poumons, Lille, France; 25 CHU de Nancy - Hôpitaux de Brabois, Nancy, France; 26 Centre de Ressources et de Compétence de la Mucoviscidose Adulte, Centre Hospitalier Jean Minjoz, Besancon, France; 27 Centre de Ressources et de Compétence de la Mucoviscidose Enfants, Hôpital d'Enfants de la Timone, Marseille, France; 28 Centre de Ressources et de Compétence de la Mucoviscidose Enfants, Hôpital Trousseau, Paris, France; 29 Centre de Ressources et de Compétence de la Mucoviscidose, CHU Estaing, Clermont-Ferrand, France; 30 Centre de ressources et de compétences pour la mucoviscidose, Hôpital des enfants, CHU Toulouse, Toulouse, France; 31 Centre de Ressources et de Compétence de la Mucoviscidose Pédiatrique, Hospices Civils de Lyon, Bron, France; 32 Centre de Référence Adulte de la Mucoviscidose, Hospices Civils de Lyon, Université de Lyon, Lyon, France; 33 Centre hospitalier régional universitaire Bretonneau, Tours, France; 34 Université de Bordeaux, CRCM pédiatrique, center de Recherche Cardio-thoracique de Bordeaux, INSERM U1045, Bordeaux Imaging Center, Bordeaux, France; 35 Laboratory of Biochemistry, Hôpital Universitaire Necker Enfants Malades AP-HP Centre, Paris, France; 36 Pharmacie Delpech, Paris, France; 37 Institut Cochin, Inserm U1016, Paris, France; 38 Respiratory Medicine and Cystic Fibrosis National Reference Center, Hôpital Cochin, AP-HP. Centre Université Paris Cité, Paris, France; 39 ERN-Lung CF network, Frankfurt, Germany; 40 Vaincre La Mucoviscidose, Paris, France; 41 IRCCS IstitutoGianninaGaslini, CysticFibrosis Center, Genoa, Italy; 42 Telethon Institute of Genetics and Medicine (TIGEM), Pozzuoli, Italy; 43 Centre de ressources et de compétences pour la mucoviscidose Adultes, Centre hospitalier régional universitaire de Nancy, Vandœuvre-Lès-Nancy, France; 44 Service de Médecine Génomique des Maladies de Système et d'Organe, Hôpital Cochin, Paris, France; 45 Laboratory of Respiratory Diseases and Thoracic Surgery, KU Leuven, Belgium; 46 Department of Physics and Biophysics, Institute of Biology, Warsaw University of Life Sciences, Warsaw, Poland

**Keywords:** cystic fibrosis, CF, CFTR (cystic fibrosis transmembrane conductance regulator), N1303K-CFTR, sweat gland, CFTR modulator, elexacaftor/tezacaftor/ivacaftor (ETI), airway epithelium

## Abstract

**Rationale:**

Respiratory status of people with Cystic Fibrosis (pwCF) carrying N1303K is improved by Elexacaftor/Tezacaftor/Ivacaftor (ETI) but, contrary to other mutations, the impact on sweat test results is limited.

**Methods:**

To explore this discrepancy, we implemented new sweat gland and respiratory cell lines stably expressing Wild type (WT)-, F508del- and N1303K-CFTR. CFTR dependent chloride (Cl^−^) and bicarbonate (HCO_3_-) transport was measured by short circuit current in these new models and in primary Human Nasal Epithelial Cells (HNECs). CFTR expression was evaluated by Western blot.

**Results:**

In the airway and the sweat gland cells expressing F508del-CFTR, ETI induced maturation of CFTR and increased Cl^−^ transport. In the respiratory cell lines and HNECs, N1303K-CFTR generated both immature and mature forms of CFTR. Correction by ETI increased CFTR amounts without promoting its maturation and improved Cl^−^ secretion. N1303K-CFTR channel activity was markedly increased by co-potentiation of IVA with Apigenin. In the sweat gland, N1303K-CFTR was expressed as a globally misfolded protein, non-rescuable by ETI. API treatment to 2 patients improved FEV1 without lowering sweat Cl- content.

**Conclusion:**

N1303K-CFTR shows tissue specific correction and suboptimal response to ETI which can be improved by API.

## Introduction

Cystic fibrosis (CF) is a life limiting autosomal genetic disease caused by bi-allelic mutations within the Cystic Fibrosis Transmembrane Conductance Regulator (*CFTR*) gene. CFTR protein absence or dysfunction and subsequent alterations induce multi-systemic damage resulting in pancreatic insufficiency, chronic bronchopathy and abnormally salty sweat (Graeber and Mall, 2023; Cutting, 2015).

The quality of life of people with CF (pwCF) was greatly improved by CFTR modulators, e.g., small molecules targeting CFTR. The most frequent variant, p. Phe508del (F508del hereafter), alters protein maturation and function (Graeber and Mall, 2023; Cutting, 2015). It can be rescued by combining correctors (VX-445, Elexacaftor, ELX, and VX-661, Tezacaftor, TEZ) that favor maturation and a potentiator (VX-770, Ivacaftor, IVA), that increases channel activity. ELX/TEZ/IVA (ETI thereafter) greatly improves the clinical status of patients carrying F508del on at least one allele (Middleton et al., 2019). Moreover, data in Fischer Rat Thyroid (FRT) cells expressing other *CFTR* variants supported the expansion of ETI approval to 271 rare CFTR mutations in the USA (Bihler et al., 2024).

p.Asn1303Lys (N1303K hereafter) represents the fourth most frequent mutation in pwCF with a relatively high allelic frequency in Italy (5.46%), France (7.9%) and Iceland (44.6%) (Prontera et al., 2016; Federici et al., 2001; Elidottir et al., 2024).

N1303K, located within the second nucleotide binding domain (NBD2), causes protein misfolding and its degradation has been reported to involve the autophagy pathway whereas F508del-CFTR is mainly degraded by the proteasome (Liu et al., 2018; He et al., 2021). Experiments in FRT cells reported that ETI restored CFTR function to 9.4% of the Wild Type (WT), approaching the 10% threshold considered predictive of clinical efficacy (Bihler et al., 2024; Durmowicz et al., 2018). Importantly, N1303K-CFTR rescue was also observed in patient-derived primary cell models (Laselva et al., 2021; Dreano et al., 2023; Huang et al., 2021; Ensinck et al., 2022) with significant effect of apigenin (API), a co-potentiator exerting synergistic effects when used in combination with IVA (Ensinck et al., 2022). Recent clinical trial and real-world data provided compelling evidence that pwCF carrying this mutation, when treated with ETI, reach significant improvement in FEV_1_, quality of life and nutrition, at a similar level as that observed for F508del pwCF (Tupayachi Ortiz et al., 2024; Solomon et al., 2024; Sadras et al., 2023; Graeber et al., 2023; Burgel et al., 2024). This supported inclusion of N1303K among the 94 additional rare variants recently approved by FDA for ETI expansion in pwCF older than 2 years (Author anonymous, 2025).

Despite these large benefits, only minimal changes in sweat chloride concentration were repeatedly observed in pwCF carrying N1303K, indicating that lung function improvement may be uncoupled from the sweat chloride reduction. At the cellular level, the reason for this discrepancy is unclear. This might include tissue specific distinct processing and degradation mechanisms, resulting in reduced expression of corrected N1303K-CFTR in the sweat gland cells as compared to lung epithelial cells. Alternatively, ETI might differently impact chloride/bicarbonate (Cl^−^/HCO_3_^−^) selectivity favoring HCO_3_^−^ over Cl^−^ transport for N1303K as compared to F508del (Zajac et al., 2023). Finally, ETI rescue of N1303K-CFTR was shown to be enhanced by the inflammatory state of the lung, a feature not present in the sweat gland (Gentzsch et al., 2024). This would lead to improvement in the lung but no major modification of Cl^−^ content in the sweat.

In this study, starting from the clinical evidence of ETI efficacy *in vivo*, we exploited patient-derived nasal epithelial cells as well as newly generated respiratory and sweat gland cell lines to investigate tissue specific *in vitro* N1303K-CFTR defects and rescue. We provide evidence of improvement of N1303K-channel activity by ETI-API combination both *in cellulo* and in patients.

## Materials and methods

Additional information is described in Supplementary Material.

### Cell models

CFF-16HBEge-CFTR cell lines expressing WT, G542X, N1303K and F508del (CFF-16HBEge-G542X, CFF-16HBEge-N1303K, CFF-16HBEge-F508del) were provided by the Cystic Fibrosis Foundation Therapeutics (Valley et al., 2019). NCL-SG3 is a human sweat gland cell line recapitulating the physiology of eccrine sweat glands established by simian virus 40 (SV40) infection of primary cultures (Lee and Dessi, 1989). NCL-SG3 cells have a mixed ductal and secretory epithelium. They express *S100A2*, *SCNN1A*, and *SSEA-4*, found in ductal cells and *SLC12A2*, encoding for the sodium-potassium-chloride cotransporter NKCC1, and *ACTA2*, both found in the acinus secretory cells (Supplementary Figure S1) (Li et al., 2017; Borowczyk-Michalowska et al., 2017). NCL-SG3 and CFF-16HBEge-G542X do not express CFTR. They were stably transduced with lentiviral vectors (gift from Dr Marianne Carlon, KU Leuven, Belgium) encoding WT, N1303K or F508del-CFTR (NCL-SG3-WT, NCL-SG3-N1303K, NCL-SG3-F508del, lenti-16HBEge-WT, lenti-16HBEge-N1303K, and lenti-16HBEge-F508del isogenic cell lines) as described previously (Bulcaen et al., 2024).

Cells were plated on 12-well plates at a density of 50,000 cells/well. One day before transduction culture medium was switched to serum-free medium to prevent interference with viral infection efficiency. Transduction was performed by overnight incubation of cells with Lentivirus particles at low MOI, followed by Puromycin selection at 1 μg/mL to generate polyclonal cell lines. Transduced cells were incubated with ELX (3 µM) and TEZ (10 µM) for 48 h to induce CFTR correction. Human Nasal Epithelial cells (HNECs) were obtained by nasal brushing and cultured as previously reported (Dreano et al., 2023; Sondo et al., 2022; Tomati et al., 2023).

### CFTR protein expression assays

CFTR expression level and profile were assessed by Western blot (Bulcaen et al., 2024; Sondo et al., 2022). Band B (core-glycosylated form) at 140 kD, band C (fully glycosylated mature form) at 180 kD, and mature intermediate forms between 140 and 180 kD were quantified by ImageJ. Band C quantification for N1303K-CFTR included the fully mature and maturation intermediates bands over 140 kD.

### Quantitative real-time PCR experiments

RNA was extracted from cell lysates using TRIzol reagent, according to the manufacturer’s recommendations (Thermo Fisher Scientific, Waltham, Massachusetts). cDNA was obtained from 200 ng of RNA by reverse transcription using RevertAid RT Kit (Thermo Fisher Scientific). Gene expression was studied using the 2-DDCT-based method and GoTaq qPCR Master Mix (Promega, Madison, Wisconsin). Each reaction was performed in triplicate and the relative expression of each gene was normalized to GAPDH expression. The primer sequences are listed in Supplementary Table S1, designed via Primerbank (pga.mgh.Harvard.edu/primerbank).

### Ussing chamber experiments

Culture inserts with differentiated cells were mounted in Ussing chambers filled with Ringer Solution and bubbled with oxygen/CO_2_ mix (Supplementary Table S2). Short-circuit current (Isc) was measured after short-circuiting the trans-epithelial ion flux with a voltage-clamp (Dreano et al., 2023; Bulcaen et al., 2024; Sondo et al., 2022). The following activators or inhibitors were added sequentially: amiloride (10 μM) at the apical side to block sodium reabsorption; CPT-cAMP (100 μM) or alternatively Forskolin/IBMX 10µM/100  μM at both apical and basolateral sides to activate CFTR; IVA (1 μM) at the apical side to potentiate the channel gate; Apigenin (API) (25 μM) at the apical side to co-potentiate IVA; and the CFTR specific inhibitor-172 (Inh-172) (20 μM) at the apical side (all from Sigma Aldrich). A Ringer without Cl^−^ was used to study HCO_3_- transport and bubbled with oxygen (Supplementary Table S2).

Isc change due to CFTR inhibition by Inh-172 after stimulation with cAMP agonists (∆Isc_inh-172_) served as an index of CFTR function. Only samples with a transepithelial resistance above 300 Ω*cm^2^ were analyzed.

### YFP-based assay for CFTR activity

CFBE41o- cells stably expressing the halide-sensitive yellow fluorescent protein (HS-YFP) were transiently transfected using Lipofectamine 2000 with vectors encoding WT, N1303K or F508del-CFTR. YFP quenching rate was used to assess CFTR activity (Sondo et al., 2022; Tomati et al., 2023).

### Patients

Thirty-six Italian patients (Ethics Committee of the Istituto Giannina Gaslini; CER 28/2020, 04/04/2020) provided HNECs by nasal brushing (Supplementary Table S3). Six French patients were recruited for HNEC sampling within an ongoing study (ClinicalTrials.gov: NCT02965326, AFSSAPS (ANSM) B1005423-40; Eudract 2010-A00392-37; CPP IDF2: 2010-05-03-3). Fifty-two French patients carrying N1303K on at least 1 allele, in *trans* with a minimal function (MF) variant non rescuable by ETI, entered the compassionate French program (Burgel et al., 2024) and were compared to 80 patients carrying F508del on at least one allele, initiating ETI, enrolled in the MODUL-CF real world French study (NCT04301856, Central IRB APHP 2019-06-12 A8) (Supplementary Table S4). French patients were evaluated before ETI administration, and at 1-month ETI for Forced Expiratory Volume, expressed in percentage predicted (ppFEV_1_) and sweat Cl^−^ concentration. Two N1303K patients were treated with Apigenin, 100 mg/kg/day, in addition to ETI. Written informed consent was obtained from each adult patient or parent for pwCF aged below 18 years.

### Statistics

Statistical analyses were performed with Statview or R. Variables were expressed as percentages or mean (Standard deviation). Comparisons were made by paired Student’s t-test, one-way analysis of variance (ANOVA) with Tukey’s or Dunett’s post-test, Wilcoxon and Mann-Whitney nonparametric tests and Repeated Measures ANOVA accordingly. Correlations were tested by Spearman test. p < 0.05 was considered significant.

## Results

### ETI rescues N1303K-CFTR chloride transport in the airways but not in the sweat glands

**FIGURE 1:**
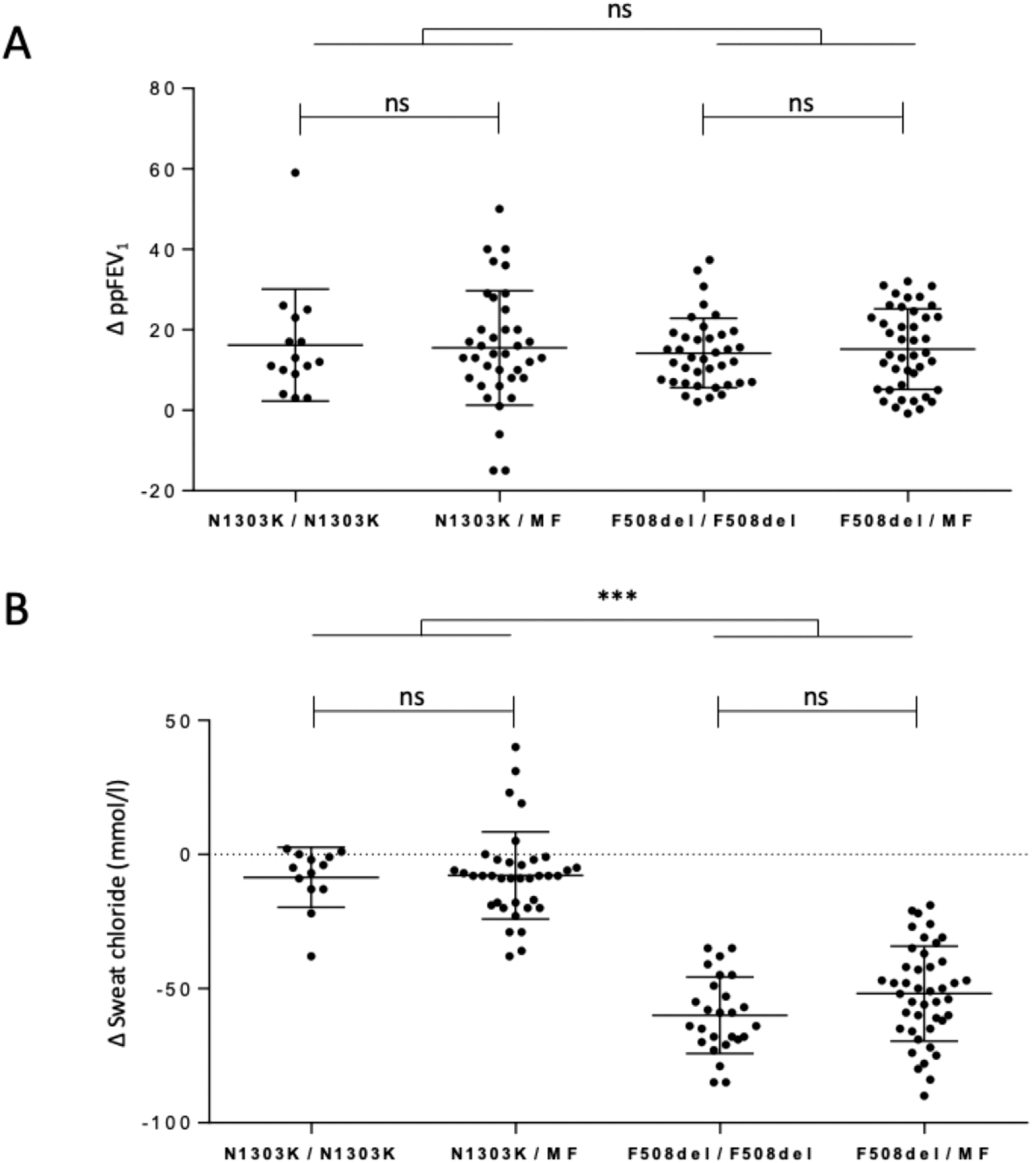
Change in percent predicted forced expiratory volume in 1 s and sweat chloride concentration in pwCF treated with Elexacaftor/Tezacaftor/Ivacaftor (ETI) carrying the N1303K or the F508del variant. Scatter dot plots indicate the corresponding variation after 2 months ETI in **(A)** percentage predicted Forced Expiratory Volume in 1 s (ΔppFEV 1) and **(B)** sweat chloride in mmol/l (Δsweat chloride). Comparison by unpaired t-test. ***: p < 0.001.

Fifty-two French patients carrying N1303K on at least 1 allele were treated with ETI. They were matched to 80 age-paired F508del patients. As expected, at 1 month, ppFEV_1_ improved by a mean of 16% ± 14%; as shown in Figure 1A (absolute difference of ppFEV_1_ at 1-month ETI versus baseline; p < 0.0001, paired t-test) a level not significantly different from the improvement observed in the 80-age paired F508del patients (15% ± 9.3%; p < 0.0001) (Supplementary Table S4). In contrast, sweat chloride concentration decreased by a mean of 8 ± 15 mmol/L in N1303K pwCF and remained elevated above 60 mmol/L in all subjects, irrespective of the genotype. Conversely, in the F508del patients, sweat test decreased by 56 ± 17.1 mmol/L (p < 0.0001for F508del versus N1303K pwCF) (Figure 1B). The changes in ppFEV_1_ or in sweat test were not significantly different between homozygotes and compound heterozygotes, either for N1303K and F508del.

### N1303K rescue in 16HBEge- and NCL-SG3 cell lines

To better understand the differential response between airways and sweat glands, we studied CFTR rescue in the novel airway and sweat gland cell models. To this aim, airway CFF-16HBEge-G542X-CFTR and sweat gland NCL-SG3 epithelial cells were transduced by lentivirus to generate cell lines stably expressing WT-CFTR, N1303K-CFTR and F508del-CFTR.

**FIGURE 2:**
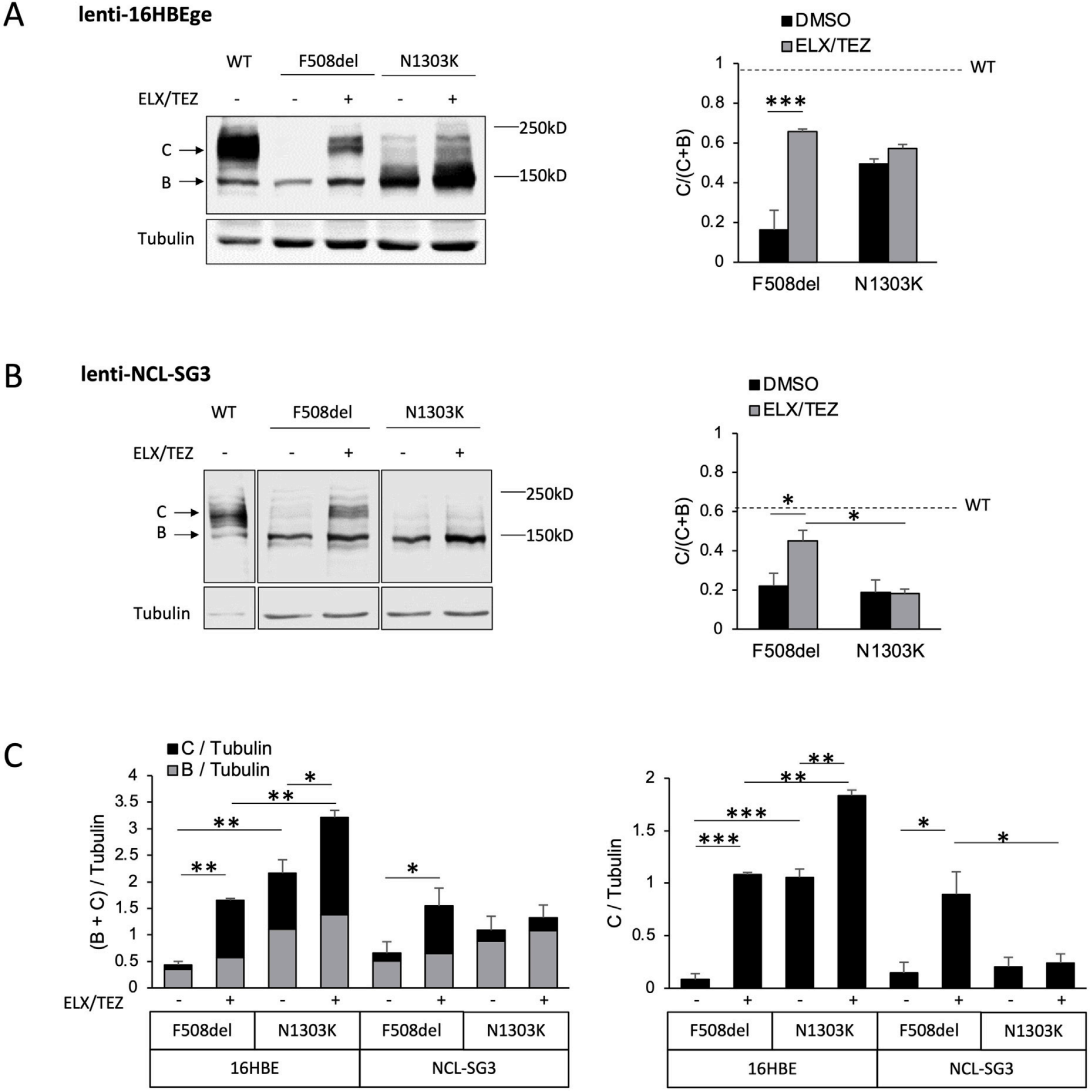
Expression of CFTR in respiratory and sweat gland cell lines stably expressing WT, F508del and N1303K-CFTR. Representative Western blot images and corresponding quantifications for WT, F508del and N1303K-CFTR in lenti-16HBEge-cells stably expressing CFTR after lentiviral transduction **(A)** sweat gland lenti-NCL-SG3 stably expressing CFTR after lentiviral transduction **(B)**. Cells were treated for 48 h with vehicle (DMSO) or ELX/TEZ combination (3 µM/10 µM). Protein detection in **(B)** was performed on a single membrane but image was fragmented and reorganized for sample order as in **(A)**. Detection of CFTR variants was performed on separate membranes and imaged separately. Bar graphs on the right panels correspond to the quantification of CFTR maturation expressed as a C/(C + **(B)** band ratio for each different cell type. As a reference, the dotted bar on the graph corresponds to the ratio for WT. **(C)** Comparison of total expression (left) and C band expression (right) of F508del and N1303K-CFTR at baseline and in TEZ/ELX corrected cells in lenti-16HBEge and lenti-NCL-SG3 cells. Data are presented as mean ± standard error (SEM) from a minimum of three independent experiments. *: p < 0.05; **: p < 0.01; ***: p < 0.001.

We first checked that CFTR transcript levels were similar for the 3 genotypes (Supplementary Figure S2). We then evaluated the CFTR protein expression pattern in the different cell models under resting condition and upon treatment with ETI. In both lenti-16HBEge- and lenti-NCL-SG3 cell lines, F508del-CFTR was expressed mainly as a band B in control conditions. ELX/TEZ increased protein maturation, as shown by the significant increase in the C/(B + C) ratio (Figures 2A–C; Supplementary Figure S3).

This was in contrast to N1303K which presented difference between the two tissues. In lenti-16HBEo- cells at basal conditions, N1303K-CFTR was detected as a strong band B, with intermediate bands between 140 and 180 kD, building a “smeared” C band with an overall significantly higher level of expression than in F508del expressing cells (p < 0.01) (Figures 2A–C; Supplementary Figure S3). This was in contrast to the lenti-NCL-SG3 cells, where N1303K-CFTR was detected mainly as an immature core-glycosylated protein (Figure 2B; Supplementary Figure S3). In both respiratory and sweat cell lines, ELX/TEZ did not promote maturation as assessed by the unchanged C/(B + C) ratio but increased the protein amount of immature and intermediate CFTR bands expressed in control conditions. As a result, N1303K-CFTR overall level of expression was significantly more abundant than that of F508del (p = 0.009) in the respiratory cell line, while remaining very low in the sweat gland cell line (4-fold less than in corrected lenti-NCL-SG3-F508del cells, p = 0.05) (Figure 2C).

**FIGURE 3:**
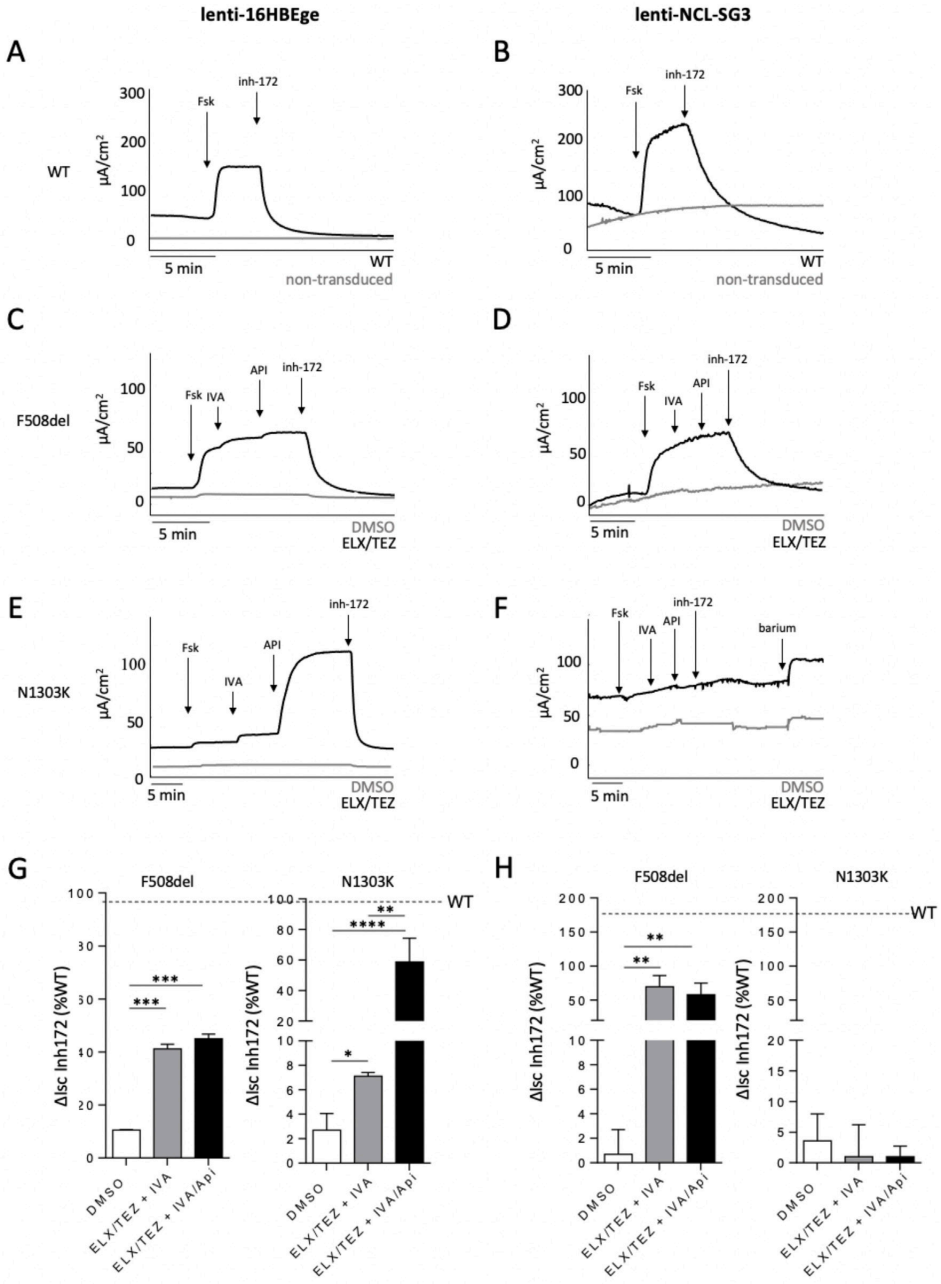
CFTR activity in respiratory and sweat gland cell lines stably expressing WT, F508del and N1303K-CFTR. CFTR activity quantified with short-circuit current technique in respiratory cell lines (lenti-16HBEge) **(A,C,E,G)** and sweat gland cell lines (lenti-NCL-SG3) **(B,D,F,H)** stably expressing WT **(A,B)**, F508del **(C,D)** and N1303K **(E,F)** after lentiviral transduction, as compared to non-transduced cells (A, B, in grey) or non-corrected epithelia (C,D,E,F, in grey). Cells were treated for 48 h with vehicle (DMSO) or ELX/TEZ combination. During the recordings, the epithelia were sequentially treated with Forskolin (Fsk) (10 µM), to activate CFTR, IVA (1 µM), to potentiate CFTR, API (25 µM), to co-potentiate IVA, CFTR inhibitor-172 (10 µM) to inhibit CFTR, all added on the apical side. Barium (5 mM) was added in lenti-NCL-SG3-N1303K on the basal side, as a quality control. Representative tracings are shown. Summary of the results in G (lenti-16HBEge) and H (lenti-NCL-SG3) from a minimum of three independent experiments. Comparison by Wilcoxon test, *: p < 0.05, **: p < 0.01; ***: p < 0.001; ****: p < 0.0001.

CFTR activity was then measured in the 2 cell models. WT-CFTR displayed a typical cAMP activated Cl^−^ transport, inhibited by Inh-172 in both sweat and respiratory cell lines (Figures 3A,B), comparable to what was observed in parental 16HBEo- cells (Supplementary Figures S4A and S4B). F508del-CFTR activity was increased by ETI in both airway and sweat gland cells, up to 29% ± 3% of the WT in the lenti-16HBEge respiratory cell lines and 44% ± 4.7% of the WT in the lenti-NCL-SG3 sweat gland cell line (p < 0.001) (Figures 3C,D,G,H).

Regarding N1303K, airway lenti-16HBEge-N1303K cells displayed a significant increase of CFTR activity in response to ETI, up to 8% ± 3.1% of the WT level. Remarkably, co-potentiation of IVA by API increased CFTR Cl^−^ transport up to 63% ± 13% of the WT (Figures 3E,G). The pattern of responses in these novel lentivirus transduced 16HBEge cell lines were similar to the ones obtained in the CFF-16HBEge-N1303K cells (Supplementary Figures S4A-D) and in the CFBE41o- cells expressing N1303K-CFTR (Supplementary Figure S5). Contrary to N1303K-CFTR, F508del-CFTR did not show API co-potentiation neither in the lenti-NCL-SG3, lenti-16HBEge-cell lines (Figures 3C,D), the CFF-16HBE (Supplementary Figure S4A), the CFBE41o- cells (Supplementary Figure S5)) nor in the HNECs from a F508del homozygous patient (Supplementary Figure S6).

In contrast to the N1303K lentivirus transduced airway epithelial cell lines, no CFTR activity was detected in the lenti-NCL-SG3-N1303K cell line neither at basal state nor after TEZ/ELX incubation or addition of API (Figures 3F,H).

### Profiles of N1303K-CFTR expression, activity and correction in primary respiratory epithelial cells

**FIGURE 4:**
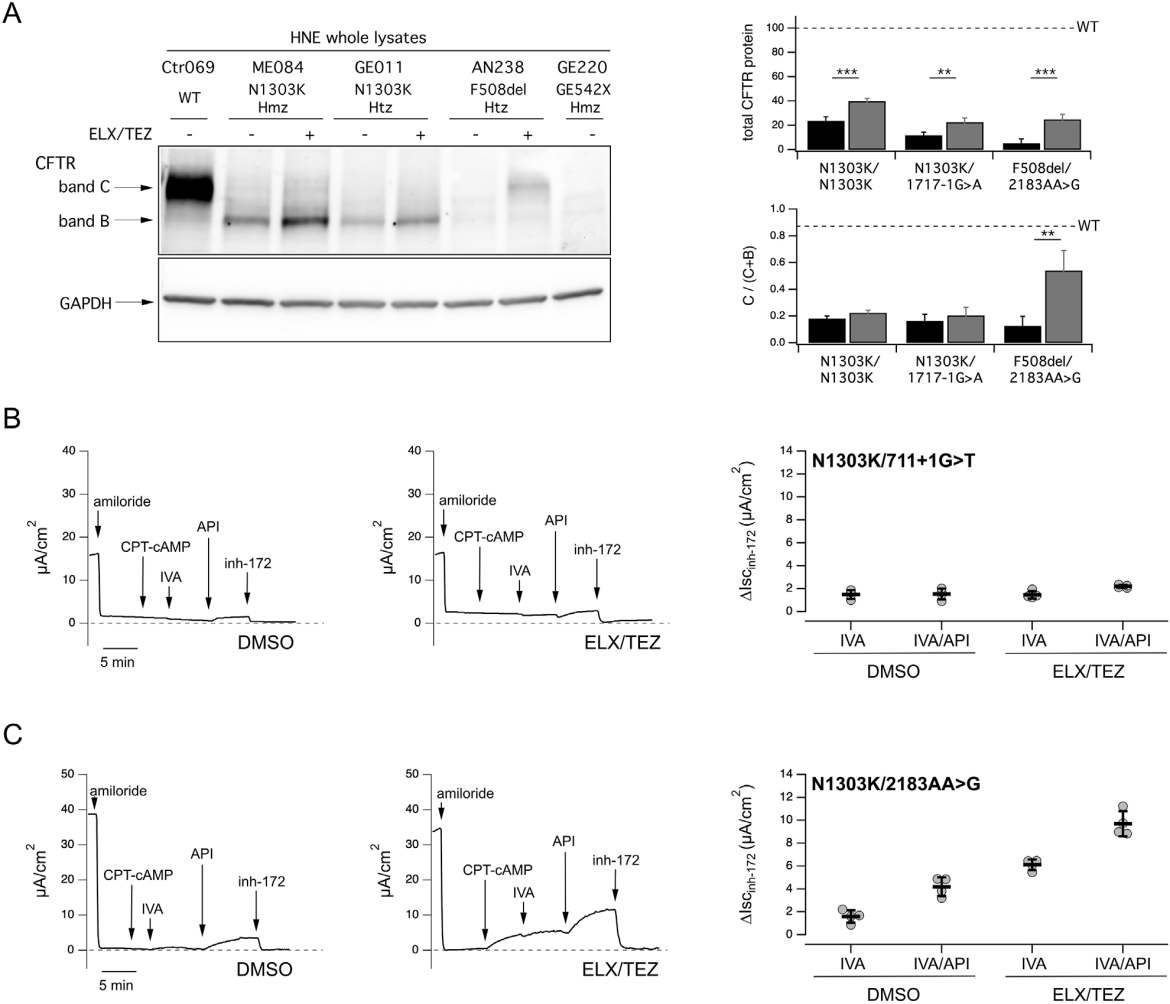
Representative biochemical and functional analysis of N1303K-CFTR in Human Epithelial Nasal Epithelial cells. **(A)** Representative Western blot images and corresponding quantifications for WT, F508del and N1303K-CFTR in Human Nasal Epithelial primary cells (HNECs). **(B,C)** Representative tracings on the left panel of the effect of vehicle (DMSO), or the elexacaftor/tezacaftor (ELX, 3 µM/TEZ, 10 µM) combination on HNECs with the short-circuit-current technique. During the recordings, the epithelia were sequentially treated (as indicated by downward arrows) with amiloride (10 μM; added on the apical side), CPT-cAMP (100 μM; added on both apical and basolateral sides), ivacaftor (IVA, 1 μM; apical side), apigenin (API, 25 μM; apical side), and the CFTR inhibitor-172 (inh-172; 20 μM; apical side). The dashed line indicates zero current level. Right panel: scatter dot plot showing the summary of results. Data reported are the amplitude of the current blocked by inh-172 (∆Isc_inh-172_). For each experimental condition the number of biological replicates were n = 4-6. Short-circuit current performed on: **(B)** N1303K/711 + 1G>T nasal epithelial cells (derived from donor ID: GE156) **(C)** N1303K/3659delC nasal epithelial cells (derived from donor ID: GE132).

In the HNECs, N1303K-CFTR was mainly detected as an immature core-glycosylated CFTR with a faint fully glycosylated mature protein. ELX/TEZ increased both band B and band C of N1303K-CFTR, but not the C/(B + C) ratio, consistent with a global increase in the protein amount but no maturation. In contrast, in F508del-CFTR cells, ELX/TEZ promoted the switch to the mature C band, indicative of improved processing and trafficking (Figure 4A).

**FIGURE 5:**
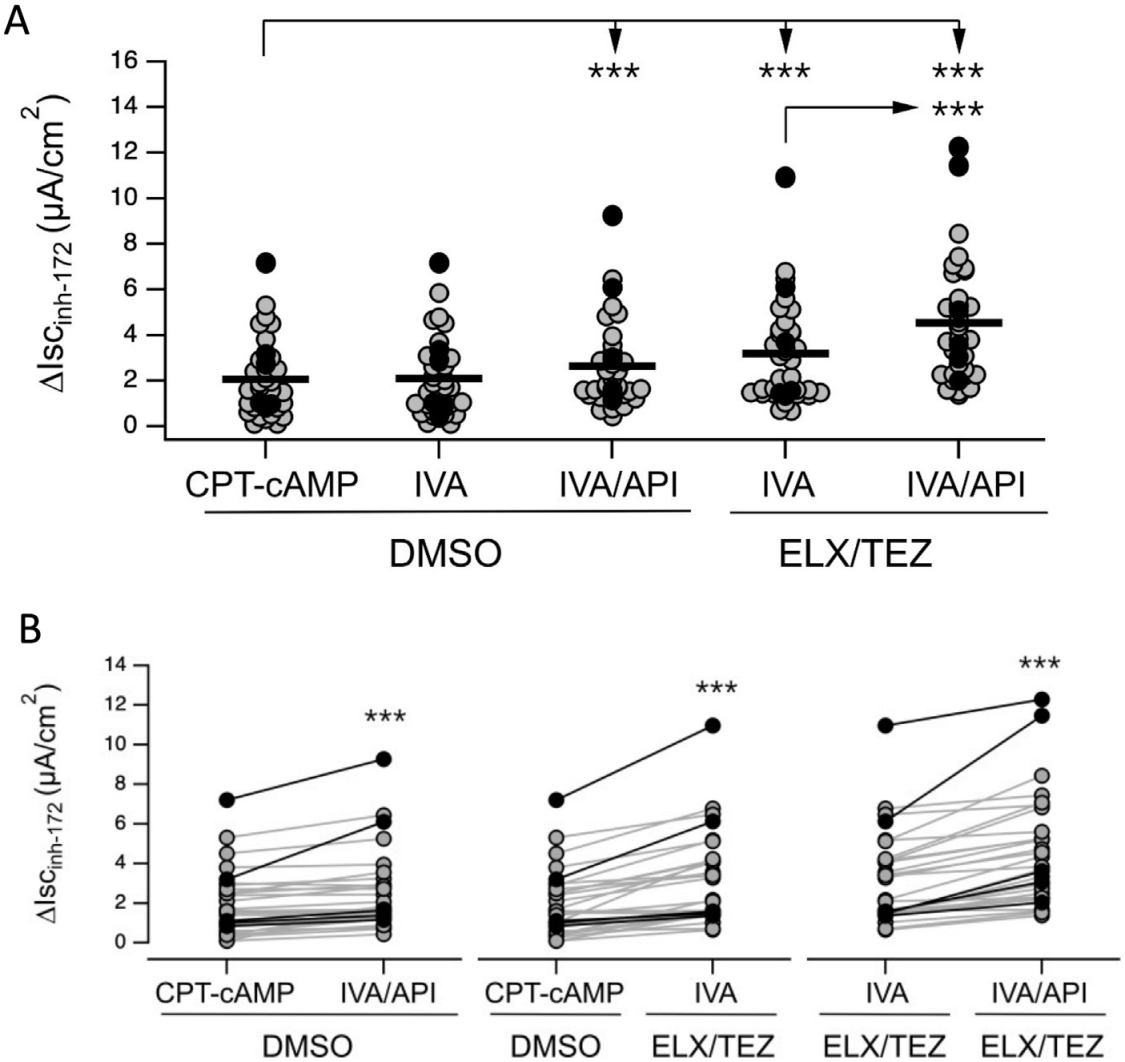
N1303K-CFTR activity potentiation by Ivacaftor and Apigenin in Tezacafor/Elexacaftor corrected Human Epithelial Nasal Epithelial cells. CFTR activity quantified with short-circuit current technique in nasal epithelial cells treated for 24 h with vehicle (DMSO) or elexacaftor/tezacaftor (ELX, 3 µM/TEZ, 10 µM) combination. During the recordings, the epithelia were sequentially treated with amiloride (10 μM; added on the apical side), CPT-cAMP (100 μM; added on both apical and basolateral sides), IVA (1 μM; apical side), API (25 μM; apical side), and the CFTR inhibitor-172 (inh-172; 20 μM; apical side). Data reported are the average amplitude of the current blocked by 20 μM inh-172 (∆Isc_inh-172_). **(A)** Scatter dot plots showing experiments performed on thirty subjects compound heterozygous for N1303K and a minimal function (MF), non-rescuable variant (N1303 K/MF) and six homozygous for N1303K (N1303K/N1303K) under different conditions. **(B)** Connected dot plots of patients shown in **(A)**.

The functional rescue of N1303K-CFTR was evaluated in HNECs from 36 Italian pwCF (Supplementary Table S4). HNECs showed different response patterns, ranging between samples with minimal basal and corrected response (Figure 4B) to samples with CFTR activity observed upon addition of IVA/API in non-corrected cells maximized by ELX/TEZ correction (Figure 4C). Results from all HNECs showed that in DMSO treated cells, average N1303K-CFTR function displayed inter-individual variability as shown in Figure 5A. CFTR activity in these non-corrected cells was not significantly increased by IVA, while addition of API after IVA enhanced CFTR activity to 9% ± 5% of the WT (p < 0.0001 versus baseline) (Figures 5A,B). Importantly, a statistically significant increase in CFTR activity in response to IVA/API was observed in one third of non-corrected HNECs samples (11/36). In ELX/TEZ-corrected HNECs, CFTR activity reached 11% ± 6% of the WT in the presence of IVA alone (ETI combination) (p < 0.0001 versus baseline) and was further increased up to 15% ± 7% of the normal when API was added to co-potentiate IVA (p < 0.0001 versus baseline; p < 0.0001 versus ETI) (Figures 5A,B). Combining IVA with API in ELX/TEZ corrected cells induced a further gain in CFTR channel activity by approximately 5%, in nearly all the samples (34/36). Importantly, in ∼ 25% of the N1303K-HNECs (9/36), CFTR activity was only improved by the quadruple cocktail ELX/TEZ/IVA/API. These values were similar to those obtained in the French patients, whose HNECs gained CFTR activity up to 10% ± 4.7% of the WT level upon ETI. This level of correction was variable and significantly correlated to their ppFEV_1_ gain at 1-month ETI of 20% ± 18% (rho = 0.781; p = 0.04) (Supplementary Table S5).

We further characterized CFTR activity by investigating CFTR-dependent HCO_3_^−^ transport. ELX/TEZ increased HCO_3_^−^ transport in F508del HNECs up to 37% ± 10% of the WT (p = 0.02; n = 4) (Supplementary Figures S7A, B, D). By contrast, N1303K-HNECs did not display a significant CFTR-dependent HCO_3_^−^ secretion neither at baseline nor upon ELX/TEZ (Supplementary Figures S7C and S7D).

To investigate whether API co potentiation of CFTR might be clinically relevant, two N1303K homozygous patients were treated with API in addition to ETI. Different attempts to generate primary nasal cells failed for the first patient. For the second patient, API co-potentiation increased CFTR activity from 16% ± 1.1% upon ETI to 43% ± 4.5% of the WT in his HNECs. At the clinical level, ppFEV_1_ of the first patient had improved from 44% to 62% of the normal at 6 months ETI. Addition of API for 2 months further increased ppFEV_1_ by 10%, and decreased sputum production but did not change sweat chloride level (103 mmol/L at API initiation, 101 mmol/L after 2 months API at 100 mg/kg/d). The patient decided to stop API because of unpleasant taste and ppFEV_1_ dropped back down to its initial value. The second patient’s ppFEV_1_ improved from 33% to ∼70% after 12 months ETI, but the patient still displayed monthly IV antibiotics. API increased ppFEV_1_ by 6% (3 different independent measurements) and decreased antibiotic needs, but did not change significantly the sweat chloride concentration (105 mmol/L at API initiation and 93 mmol/L after 2 months API). API was very well tolerated in these 2 patients.

## Discussion

Several studies performed in different cohorts of pwCF carrying N1303K show that while their respiratory status is improved by ETI, the changes in sweat chloride concentration are limited (Tupayachi Ortiz et al., 2024; Solomon et al., 2024; Sadras et al., 2023; Graeber et al., 2023; Burgel et al., 2024). We therefore aimed to investigate N1303K expression, activity and rescue in airway and sweat gland cells.

We generated new respiratory and sweat gland isogenic cell lines derived respectively from the CFF-16HBEge-G542X respiratory cell line and NCL-SG3 cells, the only human eccrine sweat gland cell line available which combines features of duct and secretory epithelia (Lee and Dessi, 1989; Li et al., 2017; Borowczyk-Michalowska et al., 2017; Servetnyk and Roomans, 2007). The use of cell lines of different tissue origin as a tool to explore differences in processing and or drug response has been successfully used in several studies (Pedemonte et al., 2010).

These novel cell models, expressing WT, F508del and N1303K-CFTR demonstrated that F508del-CFTR displayed a robust response to ETI in both tissues while N1303K-CFTR was rescued in the respiratory but not in the sweat gland cells in agreement with the minimal changes observed for sweat chloride concentration and the respiratory benefits observed in the patients.

We first asked whether ETI would preferentially restore CFTR-dependent HCO_3_^−^ transport rather than Cl^−^ transport in N1303K-CFTR expressing cells, as compared to the F508del cells. As mucin expansion is strongly dependent on HCO_3_^−^ airway content, this would improve airway mucus clearance and, as a result ppFEV_1_, but would not change the Cl^−^ sweat content (Zajac et al., 2021). This hypothesis was not confirmed since ELX/TEZ did not restore HCO_3_- transport by N1303K-CFTR in the airways, while, on the contrary, F508del-CFTR displayed a strong increase in HCO_3_^−^ transport upon ETI.

We then asked whether the observed functional discrepancy between the two tissues in patients could be linked to different level of mature CFTR. The similar CFTR transcript levels in all the cell lines suggested that the observed differences were due to post-transcriptional mechanisms. Indeed, it has been suggested that inflammation would increase the level of N1303K-CFTR rescue in the infected airways but not in sweat gland tissues which are protected from environmental aggression (Gentzsch et al., 2024). While this could indeed increase differences between the two tissues, differential responses were globally reproduced using the newly developed isogenic cell lines in absence of inflammation. This observation rather suggests that the very low level of mature N1303K-CFTR observed in the corrected sweat gland tissue explains the minimal sweat chloride correction.

Our data unraveled a specific processing defect of N1303K-CFTR in airways with both immature and maturation intermediate forms at baseline, which were increased by ELX/TEZ but did not display a maturation switch, contrary to F508del and other mutants that mature properly in response to ELX/TEZ (Veit et al., 2021a). The specific observation in around one third untreated HNECs samples of N1303K-CFTR activity if co potentiated by IVA/API indicates that these intermediate N1303K-CFTR forms can be present at the plasma membrane of airway cells in control condition and may retain biological activity.

N1303K is located in NBD2 and may hinder CFTR folding only at a late stage after partial assembly of N-terminal domains (He et al., 2021). This defect appears to be only partially rescued by ELX/TEZ type 1 correctors, which target F508del-CFTR by stabilizing MSD1 and F508del-NBD1 and promote inter-domain assembly at an earlier stage (Fiedorczuk and Chen, 2022). In this regard, adding molecules targeting the protein’s later folding stages, such as class II correctors of the NBD2/MSD2 assembly or compounds with novel mechanisms of action, could help to rescue N1303K-CFTR more effectively. In rectal organoids, corr-4a, a class II corrector, provided a small additional rescue when compared to ETI (Ensinck et al., 2022). This suggests potential for improving CFTR-N1303K maturation but the modest effect of corr-4a at the same time underlines the need to develop novel, more effective correctors to promote N1303K-CFTR maturation.

Apigenin was found to robustly increase N1303K activity but not F508del-CFTR in both respiratory cell lines and HNECs. This result is consistent with a severe gating defect of N1303K-CFTR with decreased channel open probability partially improved by IVA and further increased by IVA co-potentiation (Laselva et al., 2021; Dreano et al., 2023; Huang et al., 2021; Ensinck et al., 2022; Phuan et al., 2018; Veit et al., 2021b; Phuan et al., 2019). API potentiates CFTR mutants poorly responsive to single potentiators, possibly by binding to NBD dimer interface and stabilizing misfolded NBD2 (Moran et al., 2005). The fact that in HNECs of patients carrying N1303K, the combination of API with IVA in ELX/TEZ corrected cells induced a further gain in CFTR channel activity and the observation of a respiratory improvement in 2 patients is concordant with ETI being suboptimal for N1303K-CFTR. However, API did not change sweat chloride content, further validating our *in vitro* observations.

Limitations of the study include the fact that comparisons of CFTR protein levels and function between bronchial and sweat duct cells are based on lentivirally transduced cell lines. These models may not fully replicate tissue-specific behavior in patients. For example, both cell lines are not responsive to amiloride, an inhibitor of ENaC channels, although they express SCNN1A. NCL-SG3 cells express proteins specific for both the secretory coil and the gland duct cells, while the sweat test measures CFTR activity in the duct after metacholine sweat stimulation. Models expressing differentially coil or ductal sweat epithelium optimally from primary cells would be ideal, but are difficult to generate. As duct cells are not available, we considered that NCL-SG3 cell line expressing both secretory and ductal cell types was a good compromise. Indeed, the level of WT-CFTR activity and corrected F508del-CFTR activity was comparable to that observed in primary eccrine sweat gland culture (Eastman et al., 2024). Moreover, the fact that there is no correction even in a system overexpressing potentially CFTR, plaids for the fact that indeed N1303K-CFTR is not corrected in the sweat gland tissue at the difference of the respiratory model. This is all the more true, that even in healthy human sweat duct CFTR abundance may be low (Brown et al., 2011) depending on a variety of factors yet to be studied.

Finally, as our aim is to assess the difference between the respiratory and the sweat gland tissue, as the 2 cell lines were generated in the same manner, thanks to lentivirus overexpression, we do think that the differential level of response is scientifically relevant.

Although patients experienced beneficial clinical effect upon API, the range was not correlated to the increase in the CFTR activity observed *in vitro*. This may be explained by a potential ceiling effect for clinical improvement, and a potential cell intrinsic effect of API, not related to CFTR correction (DeRango-Adem and Blay, 2021). Moreover, the plasmatic level of API may not be sufficient to maximize the biological effect in the patient, stressing the need of optimized co-potentiator drugs (DeRango-Adem and Blay, 2021). Finally, we cannot exclude a CFTR-independent chloride transport induced by API, for ex a calcium dependent Cl^−^ efflux already reported in the NCL cell line (Servetnyk and Roomans, 2007).

The strength of the study relies on the differential study of N1303K- and F508del-CFTR combining novel airway and sweat cell lines and the largest cohort of HNECs of pwCF with N1303K studied until now. It is now important to test more mutants reported to be ETI insensitive. This is now possible thanks to the clinically relevant primary cell model. Before implementing a clinical trial, API needs to be optimized regarding PK/PD as patients did not like the bitter taste and the important number of pills to take. This will require a better understanding of the MOA of API and the selection of the mutants which can be significantly co-potentiated.

## Conclusion

Our observations, based on the largest sampling of HNECs ever studied in pwCF carrying N1303K and implementation of new respiratory and sweat gland isogenic cell lines, enabling comparison of WT, N1303K and F508del-CFTR, provide a conceptual framework of tissue dependent CFTR rescue and treatment optimization (Phuan et al., 2019). These observations pave the way to study response for variants that may be responsive to ETI in the lung but not in the sweat gland. This is particularly relevant to CF as they support approaches bringing causal therapies to pwCF with rare mutations.

## Data Availability

The original contributions presented in the study are included in the article/Supplementary Material, further inquiries can be directed to the corresponding authors.
