# Supplementary material for: Beyond Trikafta: new models to assess tissue dependent rescue of N1303K-CFTR
Source: Front Pharmacol. 2025 Oct 29;16:1661417. doi: 10.3389/fphar.2025.1661417 (PMC12605165; doi:10.3389/fphar.2025.1661417)
Supplement: Supplementary file 1 [file Image5.pdf]

# Supplemental Figure 5

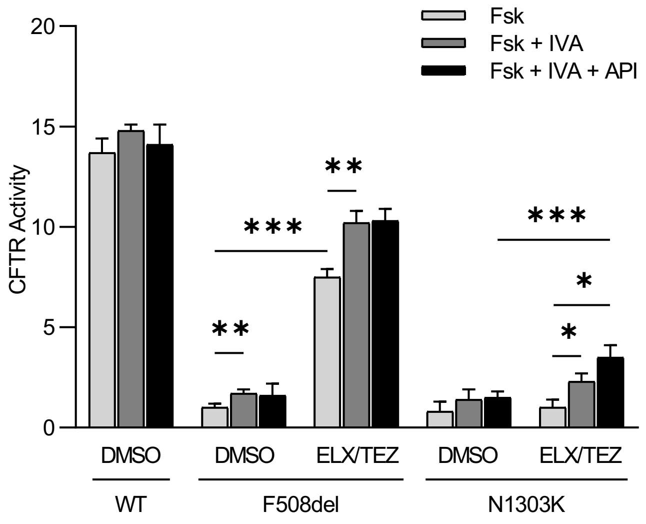


**Supplemental Figure 5. CFTR activity potentiation by Ivacaftor and Apigenin in DMSO-treated and Tezacafor/Elexacaftor corrected CFBE41o- cells**

The bar graph shows the activity of F508del and N1303K-CFTR transiently expressed in CFBE41o- cells stably expressing HS-YFP. CFTR activity was determined as a function of the YFP quenching rate following iodide influx elicited by acute incubation of Forskolin (Fsk) (20 µM) (light grey), Fsk + Ivacaftor (IVA) (1 µM) (medium grey), or Fsk + IVA + Apigenin (API) (25 µM) (dark) in cells treated for 24 h with DMSO (vehicle) or with tezacaftor (TEZ 10 µM) combined with elexacaftor (ELX 3 µM). Data from cells transiently expressing WT-CFTR (treated with DMSO) and N1303K-CFTR (treated with DMSO or ELX/TEZ) are also shown for comparison. Data expressed as mean ± standard deviation (SD) from a minimum of three independent experiments. Comparison by unpaired t-test, * for $p<0.05$, ** $p<0.005$, *** $p<0.0005$.
