# Supplementary material for: Beyond Trikafta: new models to assess tissue dependent rescue of N1303K-CFTR
Source: Front Pharmacol. 2025 Oct 29;16:1661417. doi: 10.3389/fphar.2025.1661417 (PMC12605165; doi:10.3389/fphar.2025.1661417)
Supplement: Supplementary file 2 [file Image6.pdf]

Supplemental Figure 6

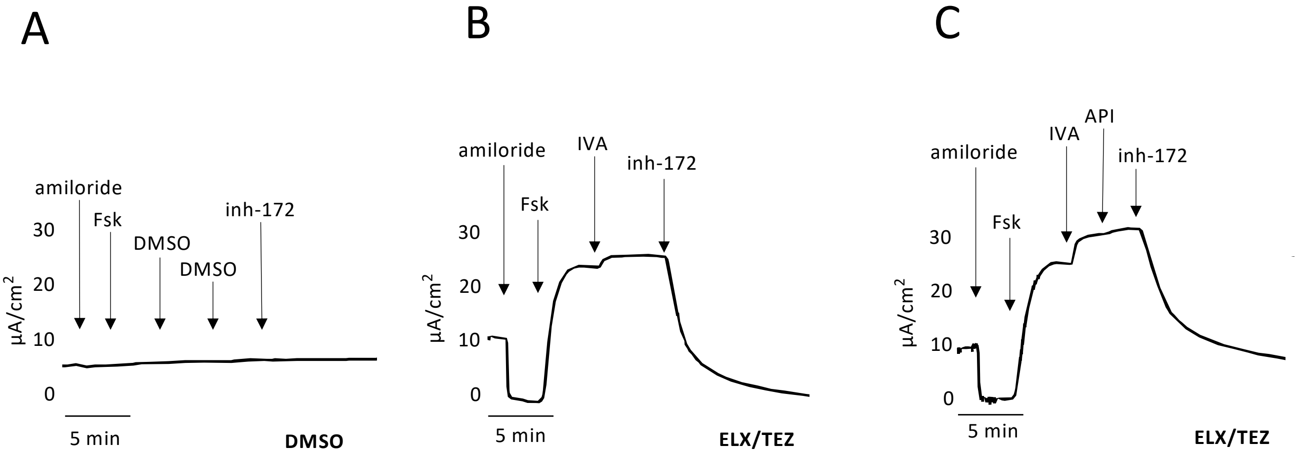

Supplemental Figure 6. CFTR potentiation by Ivacaftor and Apigenin in Tezacaftor/Elexacaftor corrected Human Nasal Epithelial cells from a F508del homozygous patient

Representative tracing of short Circuit Current (Isc) in F508del homozygous HNECs treated for 48 h with DMSO (vehicle) (A), or with Tezacaftor (TEZ)/Elexacaftor (ELX) (3 µM each) (B and C). API was added during the Ussing experiment (C). CFTR activation elicited by acute addition in the Ussing chamber of Fsk (10µM) (A), Fsk+ IVA (10 µM) (B), or Fsk + IVA + API (20 µM) (C). Three independent experiments were performed for all tested conditions.
