# Supplementary material for: Beyond Trikafta: new models to assess tissue dependent rescue of N1303K-CFTR
Source: Front Pharmacol. 2025 Oct 29;16:1661417. doi: 10.3389/fphar.2025.1661417 (PMC12605165; doi:10.3389/fphar.2025.1661417)
Supplement: Supplementary file 3 [file Supplementaryfile1.pdf]

Supplemental Materials

Authors

Iwona Pranke* & Valeria Capurro* & Benoit Chevalier* & Emanuela Pesce*, Valeria Tomati, Cristina Pastorino, Mairead Kelly-Aubert, Aurelie Hatton, Elise Dreano, Maria-Teresa Lena, Renata Bocciardi, Federico Zara, Stefano Pantano, Vito Terlizzi, Cristina Lucanto, Stefano Costa, Laura Claut, Valeria Daccò, Piercarlo Poli, Massimo Maschio, Benedetta Fabrizzi, Nicole Caporelli, Marco Cipolli, Sonia Volpi, Frederique Chedevergne, Laure Cosson, Julie Macey, Sophie Ramel, Laurence Weiss, Dominique Grenet, Laurence Le-Clainche Viala, Benoit Douvry, Bruno Ravoninjatovo, Camille Audousset, Aurélie Tatopoulos, Bénédicte Richaud-Thiriez, Melissa Baravalle, Guillaume Thouvenin, Guillaume Labbé, Marie Mittaine, Philippe Reix, Isabelle Durieu, Julie Mankikian, Stéphanie Bui,Thao Nguyen–Khoa, Karim Khoukh, Clémence Martin, Jennifer Da Silva, Paola di Carli, Carlo Castellani, Federico Cresta, Luis Galietta, Anne Guillemaut, 'Emmanuelle Girodon, Natacha Remus, Mathis Bulcaen,Marjolein Ensinck, Miroslaw Zajac, Marianne Carlon, Jean LeBihan, Pierre Régis Burgel, Isabelle Sermet-Gaudelus***# & Alexandre Hinzpeter#& Nicoletta Pedemonte***#

***co-first**

**** co second**

# co-last

Methods

Plasmids and cell models

CL-SG3 were cultured at 37 °C/5% CO_2 in William's medium-E supplemented with 5% Fetal Bovine Serum, 1% Penicillin/Streptomycin and L-Glutamine 2 mM, using standard techniques. 16HBE cells were grown at 37 °C/5% CO_2 in Minimum Essential Medium supplemented with 10% Fetal Bovine Serum and 1% Penicillin/Streptomycin (all from Thermo-Fisher Scientific, Illkirch, France).

Both, NCL-SG3 and CFF-16HBEge-G542X, were stably transduced with lentiviral vectors encoding the WT-CFTR, N1303K-CFTR or F508del-CFTR (lenti-16HBE-WT, lenti-16HBE-F508del, lenti-16HBE-N1303K) (gift from Dr Marianne Carlon, KU Leuven, Belgium). Cell lines were generated via transduction with 1/3 dilution series of vectors, overnight incubation of cells with lentiviral particles, followed by Puromycin selection (1 μg/ml).

Primary nasal epithelial cell sampling and culture

HNECs were cultured and expanded in serum-free PneumaCult Ex-Plus medium (StemCell Technologies, Vancouver, BC, Canada), supplemented with ROCK and SMAD inhibitors (Y-27632 and DMH-1, A-83-01 compounds). For the first days, culture medium also contained a mixture of different antibiotics (colistin, piperacillin, and tazobactam) to eradicate bacterial contamination. Differentiated epithelia were obtained by seeding human nasal epithelial cells (HNECs) at high density (500,000 cells/cm^2) on porous membranes (Snapwell inserts, code 3801, Corning Life Sciences, Corning, NY, USA). After 24 h, the medium was removed from both sides and replaced with Pneumacult ALI medium (StemCell Technologies, Vancouver, BC, Canada) on the basolateral side only. Epithelia differentiation (up to 16–18 days) was performed in air-liquid interface (ALI) condition.

Western Blot analysis

In brief, to remove the mucus excess, the apical side of differentiated HNEC epithelia (ALI conditions for 16 days) were washed with warm HBSS containing 0.4% sodium bicarbonate for 3 h at 37 °C. After washing twice with warm complete PBS, the apical side of the filters was dried. Basolateral culture medium (Pneumacult ALI; StemCell Technologies, Vancouver, BC, Canada) was changed to treat cells with indicated correctors or vehicle (DMSO) for 24 h at 37 °C. The following day, the newly produced mucus was removed by washing the apical

side of epithelia with warm HBSS 0.4% sodium bicarbonate at 37 °C for 30 min and then with warm complete PBS. Epithelia were lysed on ice by applying 100 µL/filter of ice-cold RIPA buffer (50 mM Tris-HCl pH 7.4, 150 mM NaCl, 1% Triton X-100, 0.5% Sodium deoxycholate, 0.1% SDS) plus proteases inhibitors (Merck KGaA, Darmstadt, Germany). Cell layers were scraped, collected in a tube, and left on ice for 15 min. To reduce the lysate viscosity, 5 × 22 G needle syringe passages followed by 5 × 27 G needle syringe passages were applied. Lysates were then cleared by centrifugation (15,000× g for 20 min at 4 °C). After centrifugation, the supernatant was transferred to a new tube and stored at −80 °C for subsequent analysis. The supernatant protein concentration was measured using a BCA assay (ThermoFisher Scientific,Waltham, MA, USA) following the manufacturer's instructions. Proteins were separated on 4–15% gradient Criterion TGX Precast gels (for HNECS epithelia) or 7% w/v acrylamide SDS-PAGE gel (for cell lines). (Bio-rad Laboratories Inc., Hercules, CA, USA) and transferred to a nitrocellulose membrane with a Trans-Blot Turbo system (Bio-rad Laboratories Inc., Hercules, CA, USA). The membranes were then incubated in a blocking buffer (3% w/v BSA (bovine serum albumin) and 3% w/v milk in PBS-0.1% v/v Tween-20) for 1 h. CFTR was detected using the mouse monoclonal anti-CFTR (ab769 and ab596, J.R. Riordan, University of North Carolina at Chapel Hill, and Cystic Fibrosis Foundation Therapeutics), diluted 1/1000 in blocking buffer while GAPDH was detected using the mouse monoclonal anti-GAPDH (sc-32233; Santa Cruz Biotechnology, Inc.), followed by either horseradish peroxidase (HRP)-conjugated anti-mouse IgG (ab97023; Abcam) or horseradish peroxidase (HRP)-conjugated goat anti-rabbit IgG (0031460; ThermoFisher Scientific, Waltham, MA, USA) and subsequently visualized by chemiluminescence using the SuperSignalWest Femto or West Dura Substrate (ThermoFisher Scientific,Waltham, MA, USA). Molecular Imager ChemiDoc XRS System (Bio-rad Laboratories Inc., Hercules, CA, USA) was used to monitor the chemiluminescence. Images were analyzed with ImageJ software (National Institutes of Health, Bethesda). The detection of the WB results was performed with the Odyssey scanner (Li-Cor, Germany). Profile analysis of different lanes was performed using the ImageJ software (National Institutes of Health, Bethesda, MD, USA) (1,2).

HS-YFP-based assay

CFBE41o- cells stably expressing the halide-sensitive yellow fluorescent protein (HS-YFP) were grown in MEM medium (Euroclone, Milan, Italy) supplemented with 10% FBS, 2 mM L-glutamine, 100 U/mL penicillin, and 100 µg/mL streptomycin (Euroclone, Milan, Italy). Vectors encoding WT-, N1303K- and F508del-CFTR variants were purchased from

VectorBuilder (vector IDs available upon request; Neu-Isenburg, Germany). For the YFP assay, 50,000 cells/well were reverse-transfected on clear-bottom 96-well black microplates (Corning Life Sciences, Corning, NY, USA) with 0.2 µg per well of the indicated vectors as previously described (3-5). In brief, cells were transfected in Opti-MEM Reduced Serum Medium (ThermoFisher Scientific, Waltham, MA, USA) using Lipofectamine 2000 (ThermoFisher Scientific, Waltham, MA, USA) as the transfection agent. Opti-MEM was carefully replaced after 6 h, with antibiotic-free culture medium. Twenty-four hours after transfection and plating, cells were treated with correctors or vehicle alone (DMSO) at the desired concentrations and incubated at 37 °C for an additional 24 h, prior to proceeding with the functional HS-YFP-based assay.

CFTR activity was determined by the HS-YFP microfluorimetric assay on CFBE41o-cells (transiently transfected to express different CFTR variants). Briefly, prior to the assay, CFBE41o- cells were washed with Dulbecco's PBS and then incubated for 25 min with 60 µL per well of Dulbecco's PBS containing forskolin (20 µM) and VX-770 (1 µM), at 37°C, to maximally stimulate the CFTR channel. Cells were then transferred to a microplate reader (Fluostar Optima; BMG Labtech, Offenburg, Germany), equipped with high-quality excitation (HQ500/20X: 500 ± 10 nm) and emission (HQ535/30M: 535±15 nm) filters for YFP (Chroma Technology, Bellows Falls, VT, USA). During the assay a continuous 14-s YFP fluorescence recording was performed, with 2 s before and 12 s after injection of 165 µL of an iodide-containing solution (Dulbecco's PBS where the NaCl was replaced by NaI; final I^- concentration 100 mM). After subtracting the background, fluorescence data were normalized to the initial value. The I^- influx rate was determined by fitting, for each well, the final 11 s of the data with an exponential function to extrapolate the initial slope (dF/dt). YFP quenching rate by I- enabled to assess CFTR activity.

Bibliography

1. Fanen P, Clain J, Labarthe R, Hulin P, Girodon E, Pagesy P, Goossens M, Edelman A. Structure-function analysis of a double-mutant cystic fibrosis transmembrane conductance regulator protein occurring in disorders related to cystic fibrosis. FEBS Lett. 1999 Jun 11;452(3):371-4. doi: 10.1016/s0014-5793(99)00647-x. PMID: 10386624.

2. Baatallah N, Elbahnsi A, Chevalier B, Castanier S, Mornon JP, Pranke I, Edelman A, Sermet-Gaudelus I, Callebaut I, Hinzpeter A. Acting on the CFTR Membrane-Spanning Domains Interface Rescues Some Misfolded Mutants. Int J Mol Sci. 2022 Dec 19;23(24):16225. doi: 10.3390/ijms232416225. PMID: 36555865; PMCID: PMC9780841.

3. Tomati V, Costa S, Capurro V, Pesce E, Pastorino C, Lena M, Sondo E, Di Duca M, Cresta F, Cristadoro S, Zara F, Galietta LJV, Bocciardi R, Castellani C, Lucanto MC, Pedemonte N. Rescue by elexacaftor-tezacaftor-ivacaftor of the G1244E cystic fibrosis mutation's stability and gating defects are dependent on cell background. J Cyst Fibros. 2023 May;22(3):525-537. doi: 10.1016/j.jcf.2022.12.005. Epub 2022 Dec 19. PMID: 36543707.

4. Terlizzi V, Pesce E, Capurro V, Tomati V, Lena M, Pastorino C, Bocciardi R, Zara F, Centrone C, Taccetti G, Castellani C, Pedemonte N. Clinical Consequences and Functional Impact of the Rare S737F CFTR Variant and Its Responsiveness to CFTR Modulators. Int J Mol Sci. 2023 Mar 31;24(7):6576. doi: 10.3390/ijms24076576. PMID: 37047546; PMCID: PMC10095403.

5. Sondo E, Cresta F, Pastorino C, Tomati V, Capurro V, Pesce E, Lena M, Iacomino M, Baffico AM, Coviello D, Bandiera T, Zara F, Galietta LJV, Bocciardi R, Castellani C, Pedemonte N. The L467F-F508del Complex Allele Hampers Pharmacological Rescue of Mutant CFTR by Elexacaftor/Tezacaftor/Ivacaftor in Cystic Fibrosis Patients: The Value of the Ex Vivo Nasal Epithelial Model to Address Non-Responders to CFTR-Modulating Drugs. Int J Mol Sci. 2022 Mar 15;23(6):3175. doi: 10.3390/ijms23063175. PMID: 35328596; PMCID: PMC8952007.
