# Supplementary material for: Beyond Trikafta: new models to assess tissue dependent rescue of N1303K-CFTR
Source: Front Pharmacol. 2025 Oct 29;16:1661417. doi: 10.3389/fphar.2025.1661417 (PMC12605165; doi:10.3389/fphar.2025.1661417)
Supplement: Supplementary file 4 [file Image4.pdf]

Supplemental Figure 4

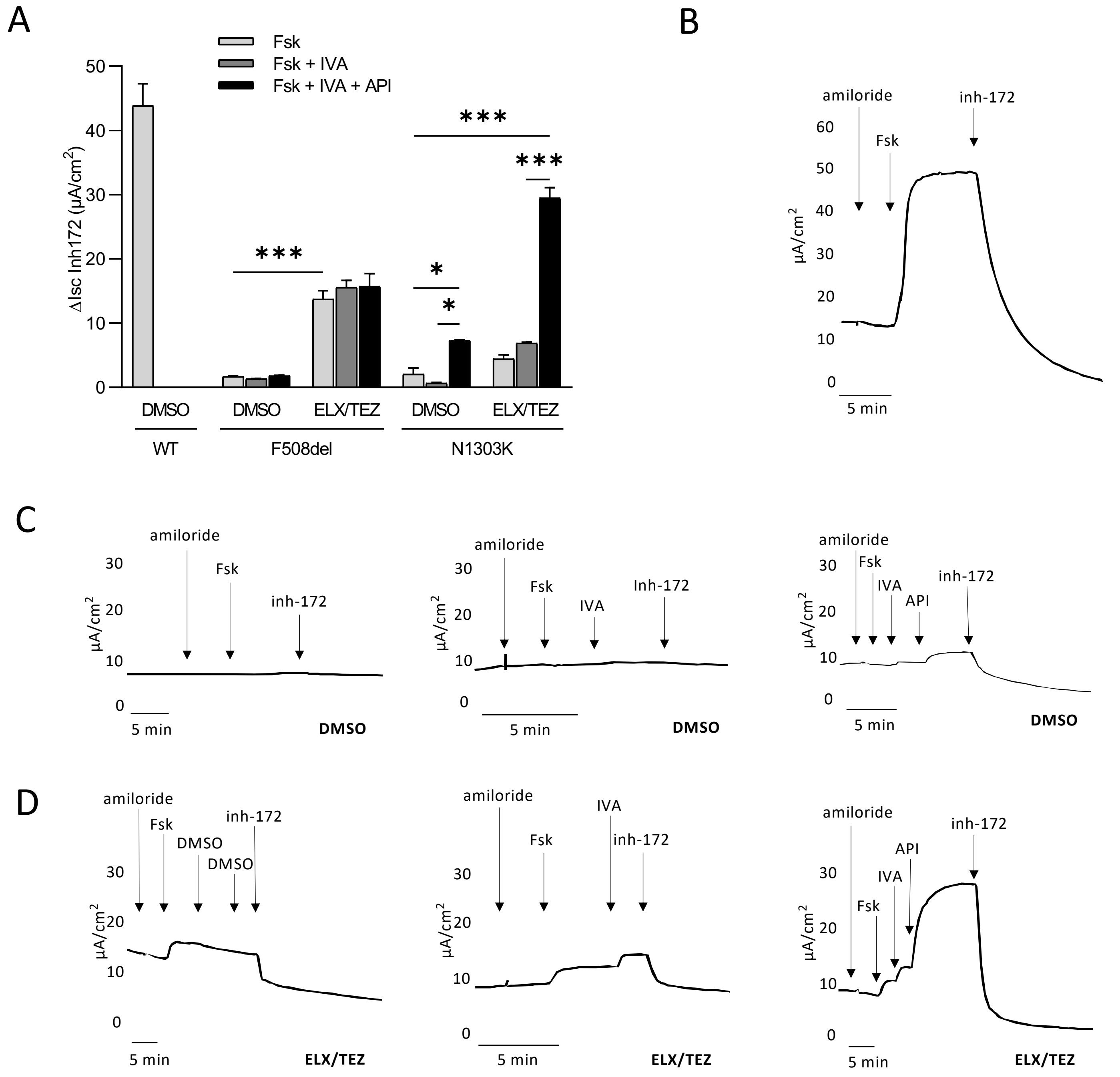

Supplemental Figure 4. CFTR activity potentiation by Ivacaftor and Apigenin in DMSO-treated and Tezacafor/Elexacaftor corrected CFF-16HBEge cells

CFTR activity in CFF-16HBEge cell lines was assessed by Short Circuit Current (Isc). CFTR activation was elicited by acute addition in the Ussing chamber of Forskoline (10 μM) (light grey), or Fsk+ IVA (10 μM) (medium grey), or Fsk + IVA + API (20 μM) (dark), in cells treated for 48 h with DMSO (vehicle), or with TEZ/ELX (3μM each).

Data from cells expressing either WT-CFTR (treated with DMSO) or F508del-CFTR (treated with DMSO or ELX/TEZ 3μM) are also shown for comparison. Experiments were performed three times and results presented as mean ± standard deviation. 2-way ANOVA statistical test, * $p<0.05$, *** $p<0.0005$.

Summary in A). Representative short-circuit currents obtained in parental 16HBEo- cells (B); N1303K-CFTR cells incubated for 48 hours with C) DMSO or D) ELX/TEZ (3 μM each).
