# Supplementary material for: Beyond Trikafta: new models to assess tissue dependent rescue of N1303K-CFTR
Source: Front Pharmacol. 2025 Oct 29;16:1661417. doi: 10.3389/fphar.2025.1661417 (PMC12605165; doi:10.3389/fphar.2025.1661417)
Supplement: Supplementary file 5 [file Image2.pdf]

Supplemental Figure 2

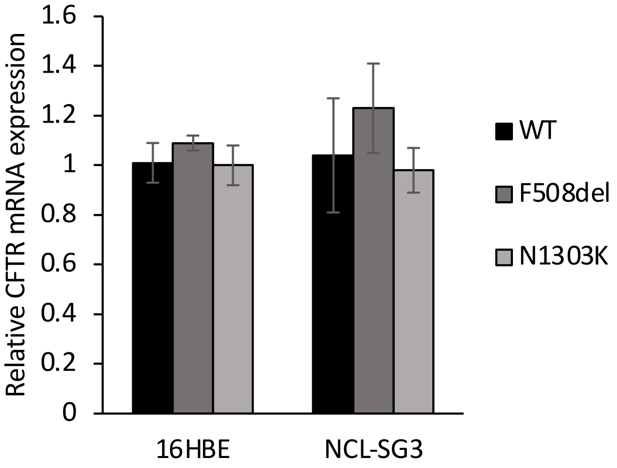

Supplemental Figure 2. CFTR transcript level in respiratory and sweat gland cell lines stably expressing WT, F508del and N1303K-CFTR

Summary data of CFTR mRNA quantification in lenti-16HBEge and lenti-NCL-SG3 cell lines expressing WT, F508del, or N1303K-CFTR after lentiviral transduction. Histogram present relative mRNA levels *versus* GAPDH obtained from 3 culture inserts for each cell line. Data presented as mean ± standard deviation of 3 technical replicates.
