# Supplementary material for: Beyond Trikafta: new models to assess tissue dependent rescue of N1303K-CFTR
Source: Front Pharmacol. 2025 Oct 29;16:1661417. doi: 10.3389/fphar.2025.1661417 (PMC12605165; doi:10.3389/fphar.2025.1661417)
Supplement: Supplementary file 6 [file Image3.pdf]

# Supplemental Figure 3

A

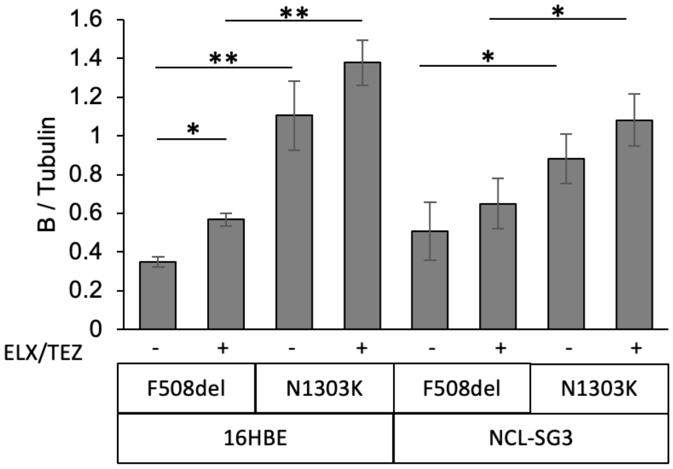


B

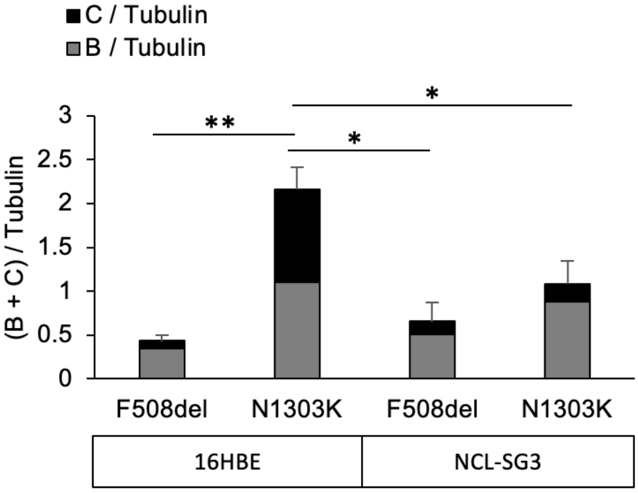


**Supplemental Figure 3. Expression of CFTR in respiratory and sweat gland cell lines stably expressing F508del and N1303K-CFTR at baseline and in Tezacafor/Elexacaftor corrected cells.**
(A) Comparison of B band expression of F508del- and N1303K-CFTR at baseline and in Tezacaftor (TEZ)/Elexacaftor (ELX) corrected lenti-16HBEge-cells and lenti-NCL-SG3 sweat gland cells stably expressing CFTR after lentiviral transduction. Cells were treated for 48 hours with vehicle (DMSO) or ELX/TEZ combination (3 μM/10 μM). Data are presented as mean ± standard error (SEM) from a minimum of three independent experiments. *: $p<0.05$; **: $p<0.01$; ***: $p<0.001$.
(B) Comparison of B + C band expression of F508del- and N1303K-CFTR normalized to tubulin level at baseline in lenti-16HBEge-cells and lenti-NCL-SG3 sweat gland cells stably expressing CFTR after lentiviral transduction. Simplified representation of data presented on Figure 2 to highlight the differences in baseline levels. Data are presented as mean ± standard error (SEM) from a minimum of three independent experiments. *: $p<0.05$; **: $p<0.01$.
