# Supplementary material for: Beyond Trikafta: new models to assess tissue dependent rescue of N1303K-CFTR
Source: Front Pharmacol. 2025 Oct 29;16:1661417. doi: 10.3389/fphar.2025.1661417 (PMC12605165; doi:10.3389/fphar.2025.1661417)
Supplement: Supplementary file 7 [file Supplementaryfile2.pdf]

Supplemental Table 1. Primers used in RT-PCR

	Fwd	Rev
**ACTA2**	AAAAGACAGCTACGTGGGTGA	GCCATGTTCTATCGGGTACTTC
**S100A2**	GCCAAGAGGGCGACAAGTT	AGGAAAACAGCATACTCCTGGA
**SCNN1A**	TCTGCACCTTTGGCATGATGT	GAAGACGAGCTTGTCCGAGT
**SLC12A1**	CTCCTCGTCGGCTGAAGAC	CTGTGTGCCGGTAGTGATCG
**SSEA-4**	TGGACGGGCACAACTTCATC	GGGCAGGTTCTTGGCACTCT

Supplemental Table 2. Ringer composition for Ussing chamber experiments

Ussing chamber experiments		
**Bicarbonate-free condition**		**Chloride-free condition**
Apical Ringer	Basolateral Ringer	Apical and basolateral Ringer
Na Gluconate 145 mM	NaCl 145 mM	Na isethionate 125mM
K_2HPO_4 3.3mM	K_2HPO_4 3.3mM	$NaHCO_3$ 25mM
Hepes 10 mM	Hepes 10mM	K_2HPO_4 2.4mM
$CaCl_2$ 1.2 mM	$CaCl_2$ 1.2mM	KH_2PO_4 0.6mM
$MgCl_2$ 1.2mM	$MgCl_2$ 1.2mM	Ca Gluconate 3mM
D-Glucose 10 mM	D-Glucose 10mM	Mg Gluconate 2.4mM
		D-Glucose 10mM

Supplemental Table 3. Genotype of the Italian subjects.

Thirty patients compound heterozygous for N1303K and a MF, non-rescuable *CFTR* variant resembling a null allele and six patients homozygous for N1303K were included in this study. One healthy subject (donor ID: Ctr069) and one patient heterozygous for F508del and 2183AA>G (donor ID: AN238), were enrolled as further controls.

Donor ID	Allele 1	Allele 2
ME084	N1303K	N1303K
ME087	N1303K	N1303K
GE207	N1303K	N1303K
MI239	N1303K	N1303K
ME275	N1303K	N1303K
MI291	N1303K	N1303K
GE010	N1303K	394delTT
GE011	N1303K	1717-1G>A
GE018	N1303K	711+1G>T
FI034	N1303K	G542X
FI047	N1303K	1717-1G>A
FI053	N1303K	Delexon22-24
FI057	N1303K	E585X
FI064	N1303K	1717-1G>A
GE068	N1303K	W1282X
ME088	N1303K	R553X
ME090	N1303K	I507del
ME092	N1303K	G542X
ME094	N1303K	G542X
ME119	N1303K	3659delC
GE132	N1303K	2183AA>G
FI134	N1303K	1717-1G>A
GE144	N1303K	G542X
GE156	N1303K	711+1G>T
ME163	N1303K	1002-1113_1002-1110delGAAT
GE226	N1303K	1713del
MI240	N1303K	L732X
MI247	N1303K	G542X
MI248	N1303K	4016insT
MI258	N1303K	711+5G>A
MI259	N1303K	C276X
MI264	N1303K	852del22bp
GE293	N1303K	G542X
GE326	N1303K	G542X
GE331	N1303K	1717-1G>A
AN384	N1303K	Q1012X

Supplemental Table 4. Genotype of the French patients

Patient	CFTR variant	CFTR variant
	**Allele 1**	**Allele 2**
	**N1303K cohort**	
**1**	N1303K	N1303K
**2**	N1303K	N1303K
**3**	N1303K	N1303K
**4**	N1303K	N1303K
**5**	N1303K	N1303K
**6**	N1303K	N1303K
**7**	N1303K	N1303K
**8**	N1303K	N1303K
**9**	N1303K	N1303K
**10**	N1303K	N1303K
**11**	N1303K	N1303K
**12**	N1303K	N1303K
**13**	N1303K	N1303K
**14**	N1303K	N1303K
**15**	N1303K	N1303K
**16**	N1303K	R1162X
**17**	N1303K	R1162X
**18**	N1303K	G542X
**19**	N1303K	CFTRdele3-10,14b-16
**20**	N1303K	H199R
**21**	N1303K	2789+5G>A
**22**	N1303K	1898+5G>A
**23**	N1303K	4326delTC
**24**	N1303K	M1V
**25**	N1303K	711+1G>T
**26**	N1303K	E585X
**27**	N1303K	R553X
**28**	N1303K	R553X
**29**	N1303K	W1282X
**30**	N1303K	3120+1G>A
**31**	N1303K	3659delC
**32**	N1303K	3600G>A
**33**	N1303K	CFTRdele4-21
**34**	N1303K	l507del
**35**	N1303K	1677delTA

**36**	N1303K	Y1092X
**37**	N1303K	1717-1G>A
**38**	N1303K	1717-1G>A
**39**	N1303K	Q290X
**40**	N1303K	711+1G>T
**41**	N1303K	G542X
**42**	N1303K	Q493X
**43**	N1303K	3120+1G>A
**44**	N1303K	2640delT
**45**	N1303K	2789+5G>A
**46**	N1303K	1811+1,6kbA>G
**47**	N1303K	G542X
**48**	N1303K	2789+5G>A
**49**	N1303K	4374+1G>A
**50**	N1303K	G542X
**51**	N1303K	2622+1G>A
**52**	N1303K	G542X
	F508del cohort	
**53**	F508del	F508del
**54**	F508del	F508del
**55**	F508del	F508del
**56**	F508del	F508del
**57**	F508del	F508del
**58**	F508del	F508del
**59**	F508del	F508del
**60**	F508del	F508del
**61**	F508del	F508del
**62**	F508del	F508del
**63**	F508del	F508del
**64**	F508del	F508del
**65**	F508del	F508del
**66**	F508del	F508del
**67**	F508del	F508del
**68**	F508del	F508del
**69**	F508del	F508del
**70**	F508del	F508del
**71**	F508del	F508del
**72**	F508del	F508del
**73**	F508del	F508del
**74**	F508del	F508del
**75**	F508del	F508del
**76**	F508del	F508del
**77**	F508del	F508del
**78**	F508del	F508del
**79**	F508del	F508del
**80**	F508del	F508del
**81**	F508del	F508del
**82**	F508del	F508del
**83**	F508del	F508del

**84**	F508del	F508del
**85**	F508del	F508del
**86**	F508del	F508del
**87**	F508del	F508del
**88**	F508del	F508del
**89**	F508del	F508del
**90**	F508del	F508del
**91**	F508del	Q2X ; R3W
**92**	F508del	1717-1G>A
**93**	F508del	3659delC
**94**	F508del	1078delT
**95**	F508del	c.870-1113_870-1110del
**96**	F508del	3120+1G>A
**97**	F508del	3532AC>GTA
**98**	F508del	CFTRdele2,3
**99**	F508del	W1282X
**100**	F508del	K1165X
**101**	F508del	R1162X
**102**	F508del	394delTT
**103**	F508del	3120+1G>A
**104**	F508del	N1303K
**105**	F508del	574delA
**106**	F508del	3121-1G>A
**107**	F508del	G745X
**108**	F508del	CFTRdele2
**109**	F508del	3120+1G>A
**110**	F508del	I1005R
**111**	F508del	3659delC
**112**	F508del	R785X
**113**	F508del	675del4
**114**	F508del	R553X
**115**	F508del	Y122X
**116**	F508del	CFTRdele2,3
**117**	F508del	E60X
**118**	F508del	A559T
**119**	F508del	2747delC
**120**	F508del	H199Y
**121**	F508del	E60X
**122**	F508del	Y1092X(C>A)
**123**	F508del	W846X
**124**	F508del	CFTRdele2,3
**125**	F508del	G85E
**126**	F508del	I507del
**127**	F508del	G542X
**128**	F508del	N1303K
**129**	F508del	Y122X
**130**	F508del	CFTRdele16-17a-17b
**131**	F508del	R75X
**132**	F508del	N1303K

Supplemental Table 5. Clinical response to ETI of N1303K French patients and CFTR function improvement upon ETI measured *in vitro* in their HNECs.

N° Patient	Allele 1	Allele 2	ΔppFEV1 ETI	ΔST ETI (mmol/l)	ΔFsk/IBMx ETI (% of WT)	ΔInh172 ETI (% of WT)
6	N1303K	N1303K	11	-	10	8,6
10	N1303K	N1303K	13	-2	6,2	6,5
5	N1303K	N1303K	10	-5	5	19
2	N1303K	N1303K	37	-9	16,7	15,8
28	N1303K	711+1G>T	10	31	3,3	7,7
19	N1303K	R1162X	29	-9	9	8,3
22	N1303K	CFTRdele3-10,14b-16	25	-8	10	6
21*	N1303K	G542X	2	-8	8,2	7,5

*Additional genetic investigations for this non responder patient did not find a complex allele in *CFTR* but identified a mutation in *SCNN1A* (R181W), which increases ENaC activity, as suggested by the response to Amiloride at 76µA/cm^2 observed in this patient (3.8 fold of that observed in other HNECs carrying N1303K)
