# Supplementary material for: Beyond Trikafta: new models to assess tissue dependent rescue of N1303K-CFTR
Source: Front Pharmacol. 2025 Oct 29;16:1661417. doi: 10.3389/fphar.2025.1661417 (PMC12605165; doi:10.3389/fphar.2025.1661417)
Supplement: Supplementary file 8 [file Image7.pdf]

Supplemental Figure 7

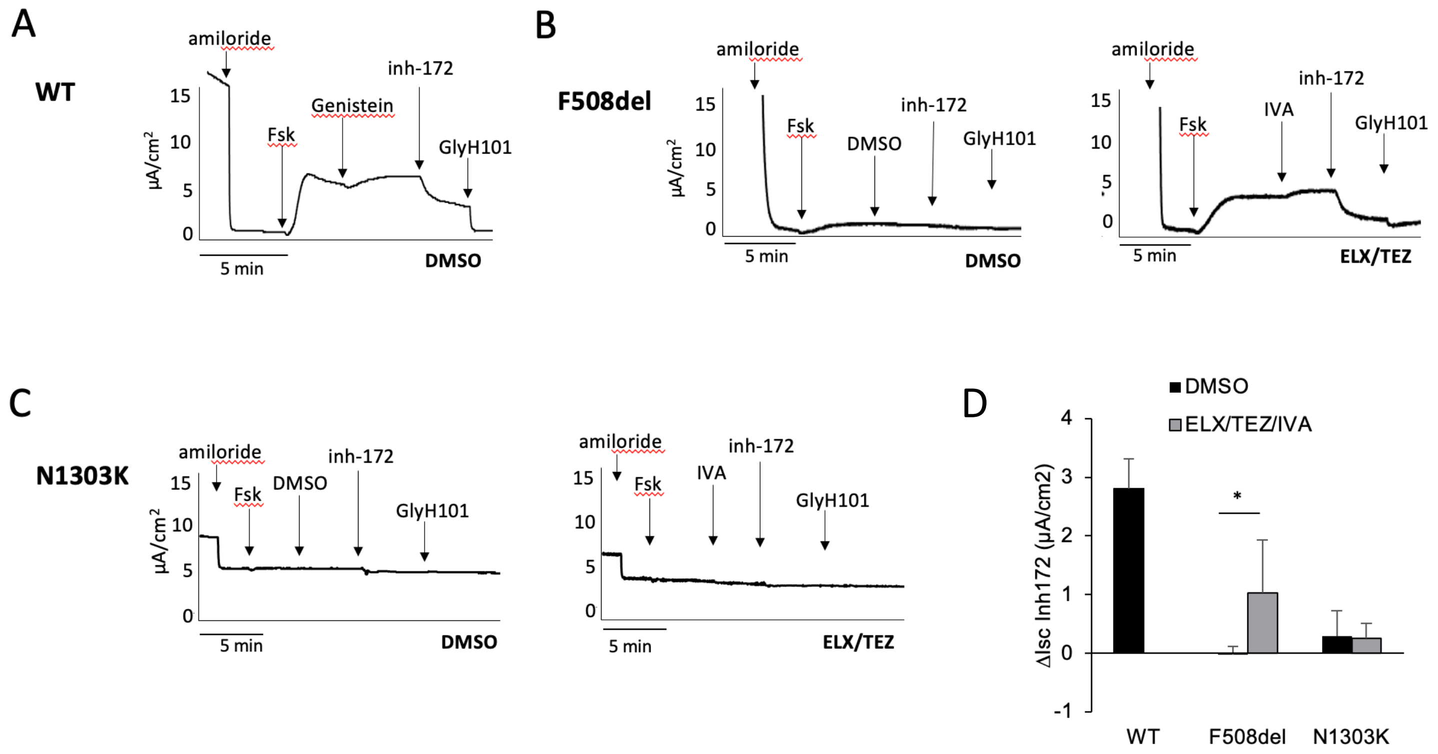

Supplemental Figure 7. Bicarbonate transport in Human Nasal Respiratory Epithelial cells expressing WT, F508del or N1303K-CFTR.

Bicarbonate secretion was quantified with Short-circuit current technique in Human Nasal Respiratory Epithelial cells (HNECs) treated for 48 hours with vehicle (DMSO) or ELX/TEZ combination. During the recordings, the epithelia were treated with amiloride (10 µM; added on the apical side). CFTR activation was elicited by acute addition in the Ussing chamber of Forskoline (Fsk) (10 µM), or Fsk+ IVA (1 µM), or Fsk + IVA + API (25 µM), followed by inhibition by inhibitor-172 (Inh-172; 10 µM; apical side) and GlyH-101 (10 µM; apical side). Representative tracings obtained in respiratory HNECs from WT (A), F508del homozygous (B) and N1303K homozygous (C) patients. Cells were treated with DMSO (left side graphs) or ELX/TEZ (right side graphs). (D) Data expressed as mean ± standard deviation (SD) obtained from 4 different WT, 4 F508del+/+ and 4 N1303K+/+ patient's HNECs. Mann-Whitney test * < 0.05.
