# Supplementary material for: Beyond Trikafta: new models to assess tissue dependent rescue of N1303K-CFTR
Source: Front Pharmacol. 2025 Oct 29;16:1661417. doi: 10.3389/fphar.2025.1661417 (PMC12605165; doi:10.3389/fphar.2025.1661417)
Supplement: Supplementary file 9 [file Image1.pdf]

Supplemental Figure 1

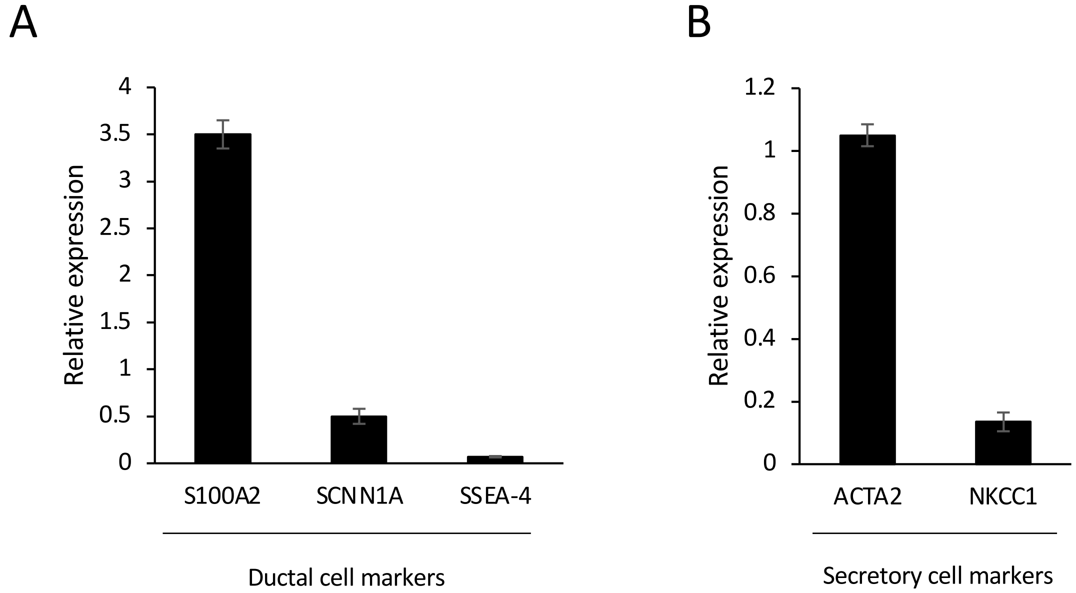

Supplemental Figure 1. NCL-SG3 cell lines express genes of ductal and secretory coil cells

Relative mRNA expression normalized to GAPDH expression in lenti-N1303K-NLC-SG3 cells of ductal cell markers (*S100A2*, which encodes for S100A2, *SCNN1A*, which encodes for the sodium channel epithelial 1 subunit alpha, and *SSEA-4*, which encodes for stage-specific embryonic antigen-4) and secretory cell markers (*SLC12A2*, which encodes for the Na-Cl-K transporter and *ACTA2*, which encodes for actin alpha 2, smooth muscle). Data presented as mean ± standard deviation of 3 technical replicates.
